# Aberrant immunity in the oral cavity—a link with rheumatoid arthritis?

**DOI:** 10.3389/froh.2024.1430886

**Published:** 2024-06-14

**Authors:** Jennifer Malcolm, Shauna Culshaw

**Affiliations:** Oral Sciences, University of Glasgow Dental School, School of Medicine, Dentistry and Nursing, College of Medical, Veterinary and Life Sciences, University of Glasgow, Glasgow, United Kingdom

**Keywords:** periodontitis, rheumatoid arthritis, autoimmunity, *Porphyromonas gingivalis*, autoantibody

## Abstract

There are well established epidemiological links between rheumatoid arthritis and periodontitis. Recent data have started to shed light on the mechanisms that might underlie the relationship between these two complex diseases. Unravelling the roles of distinct pathways involved in these mechanisms has the potential to yield novel preventative and therapeutic strategies for both diseases. Perhaps most intriguingly, this represents an area where understanding the biology in the oral cavity might reveal fundamental advances in understanding immune regulation and the relationships between the host and microbiome. Here we seek to discuss aspects of the adaptive immune response that might link periodontitis and rheumatoid arthritis.

## Introduction

Autoimmune diseases predominantly develop in individuals with genetic susceptibility after exposure to environmental factors; suggesting gene:environment interactions are necessary to drive loss of immune tolerance, and emergence of pathological adaptive autoimmune responses to host tissues. However, it is increasingly clear that autoreactive CD4+ T cell and autoantibody responses can develop consequent to chronic inflammatory or metabolic disturbances, even in the absence of defined genetic susceptibility (1–3). Further, evidence is emerging that environmental exposures and environment: gene interactions driving autoimmunity may be temporally and functionally distinct (4–6). This raises the possibility that adaptive autoreactivity emerges consequent to environmental exposures as a component of chronic inflammation; and favours the progression to autoimmune disease only in genetically susceptible individuals.

Rheumatoid arthritis (RA) is a joint destructive autoimmune disease associated with adaptive immune responses towards post-translationally modified self-proteins. This autoreactivity can be readily detected as autoantibodies reactive with self-proteins containing citrulline modifications, known as ACPA. Importantly, ACPA can emerge asymptomatically in patient sera many years before the onset of joint inflammation, and strongly associate with chronic inflammatory insults at mucosal surfaces, such as smoking and periodontitis (5, 7). In people expressing human leukocyte antigen (HLA) class II alleles containing an amino acid motif known as the shared epitope [HLA-SE, reviewed (8)], this initial ACPA response can evolve and mature; a process associated with the transition to symptomatic joint inflammation. This is supported by recent data revealing that HLA-SE alleles are required for the transition from ACPA positivity to ACPA positive RA (as is widely used in the literature, in the following discussion, ACPA positivity/ACPA positive RA refers to seropositivity) (5, 6). A corollary to these data is that the pathways predisposing to ACPA positivity can occur independently of HLA-SE risk alleles following exposure to relevant environmental insults, and only predispose to autoimmune disease in genetically susceptible individuals. This distinction suggests that autoreactivity secondary to chronic inflammation or infection precipitates autoimmune responses that may either progress, or not progress, to autoimmune disease based on genetic susceptibility.

Here we review recent developments related to the impact of gene: environment interactions in the pathogenesis of rheumatoid arthritis. Using these new developments as a framework, we discuss the implications for understanding mucosal inflammatory disease and risk for RA, using periodontitis as an exemplar. Finally, we review data derived from recent mechanistic studies that offer clues and new hypotheses to determine how aberrant inflammation in the oral cavity predisposes to the development of RA.

## The distinction between ACPA positivity and ACPA positive RA

Retrospective serological studies of patients with RA reveal the presence of ACPA many years before the onset of clinically evident joint inflammation (4, 9, 10). Smoking is the strongest environmental risk factor for ACPA positivity and progression to ACPA positive RA (11). In a large Swedish twin study, smoking was associated with ACPA positivity, but APCA positive RA was associated with smoking only in individuals expressing HLA-SE (HLA-shared epitope) risk alleles (12). This observation that smoking, not HLA-SE, confers risk for ACPA positivity, while smoking in an HLA-SE background confers risk for ACPA positive RA was replicated in a meta-analysis (6). Subsequent studies have revealed that ACPA's in ACPA positive RA show somatic hypermutations, as measured by variable-domain glycosylation. This ACAP maturation is dependent on HLA-SE (13). Glycosylation of IgG ACPA is largely absent from ACPA positive individuals who do not progress to RA (14). Notably, somatic hypermutation is a T-cell dependent process—T cells help B cells to generate an array of antibody variants, with selection for those antibodies with highest affinity. Therefore, HLA-SE-restricted antigen-presentation to CD4+ T cells is implicated in the transition from ACPA positivity to ACPA positive RA (5, 13).

## Mucosal origins of ACPA

Identification of smoking as the predominant environmental risk factor for ACPA positive RA, together with observations that ACPA emerge in patients sera up to ten years before the development of joint inflammation, have led to the hypothesis that ACPA are triggered at mucosal surfaces subject to chronic inflammatory insults (15, 16). In support of this mucosal origin hypothesis is the finding that diverse mucosal inflammatory diseases are associated with elevated ACPA positivity compared with healthy individuals (Table 1). How ACPA are triggered, and the biological function of ACPA at mucosal surfaces remain unclear. It is possible that ACPA could represent a biomarker for shared inflammatory pathways occurring at distinct mucosal surfaces, in response to diverse environmental exposures.

**Table 1:** Association of ACPA positivity with mucosal exposures in patients without RA.

Reference	Disease/biomarker	% positive (unless otherwise described)			
		IgG Anti-CCP	IgA Anti-CCP	Anti-CEP	Anti-REP
(17)	Healthy controls (*n* = 30)	0	N/A	Significantly higher titres in periodontitis compared with healthy controls.	Significantly higher titres in periodontitis compared with healthy controls.
	Periodontitis (*n* = 39)	7.7	N/A		
	*P. gingivalis* positive (*n* = 16)	18.8	N/A		
	*P. gingivalis* negative (*n* = 23)	0	N/A		
(18)	Healthy controls (*n* = 98)	1	N/A	3	2
	Periodontitis (*n* = 96)	1	N/A	12	16
(19)	Healthy controls (*n* = 36)	5.6	8.3	N/A	N/A
	Periodontitis (*n* = 114)	13	16	N/A	N/A
	Bronchiectasis (*n* = 80)	21	10	N/A	N/A
	Cystic Fibrosis (*n* = 41)	24	27		
	Rheumatoid arthritis (*n* = 86)	86	74		
(20)	Healthy controls (*n* = 36)	5.6	2.8	N/A	N/A
	Ulcerative colitis (*n* = 227)	13	37		
	Crohn's disease (*n* = 164)	17	32		
	Rheumatoid arthritis (*n* = 86)	86	74		
(21)	Healthy controls (*n* = 39)	2.6	N/A	N/A	N/A
	Tuberculosis (*n* = 47)	32			
(22)	Healthy controls (*n* = 18)	6	N/A	N/A	N/A
	Tuberculosis (*n* = 49)	32			
(23)	Healthy controls (*n* = 237)	0.4	N/A	N/A	N/A
	Tuberculosis (*n* = 89)	6.7			

(24) found **no** evidence of ACPA positivity in several other infections/infectious diseases, including Mycoplasma, Toxoplasma, Salmonella, Malaria, Leishmaniasis.

N/A, Not applicable.

ACPA positivity in individuals without arthritis is associated with the presence of mucosal inflammatory exposures, including smoking, but also exposure to dysbiotic microbial communities in the gingival and intestinal tissues (Table 1). This suggests that the disease processes that give rise to ACPA, and subsequently predispose to the development of ACPA positive RA in genetically susceptible individuals are active at mucosal surfaces subject to chronic inflammatory insults. In both periodontitis and inflammatory bowel diseases, the homeostatic relationship between host-tissues and the resident polymicrobial communities in health is lost, leading to immune-mediated tissue damage driven by dysregulated host immune responses to dysbiotic polymicrobial communities. Thus, in both diseases, environment and/or genetic factors change the interaction between host-tissues and resident polymicrobial communities, leading to a break down in mucosal barrier function. It has been posited that the mucosal IgA ACPA response may have a protective role in binding and neutralising citrullinated proteins released during NETosis, and that systemic IgG ACPA responses develop consequent to breached mucosal barrier function (15). In support of this hypothesis, it was recently demonstrated that people with both RA and periodontitis can suffer repeated breaches in mucosal barrier function, leading to oral bacteraemia's and changes in circulating monocyte populations. Further, the authors revealed that resident bacteria in the mouth can be externally citrullinated (presumed to be the result of NETosis) and that hypermutated ACPA derived from RA plasmablasts can bind citrullinated oral bacteria (25).

## Oral mucosal origins of ACPA

As indicated above, the gingival tissues are a site of potential ACPA induction. Serological studies of patients with periodontitis reveal small, but measurable, serum ACPA (Table 1). Whilst the autoantibody profile in patients with periodontitis is variable, and the titres are relatively low, the disease itself is common, and the autoreactivity is greater than observed in healthy controls. Autoantibodies against both citrullinated (anti-CCP) and native forms of proteins (specific for both host and microbial) have been observed. As such, there is evidence of an adaptive immune response to microbial antigens and antigens that share homology with human proteins, such as α-enolase, and host proteins such as vimentin and fibrinogen (26). It is noteworthy that only very few infectious diseases appear to result in anti-CCP immunity. Auto-antibodies against both native and citrullinated peptides have been documented in patients with TB (with reports varying between around 6% of patients up to 37% of patients). There are small studies reporting anti-citrullinated peptide immunity in leishmaniasis, and some in hepatitis C, Lyme disease, Chagas disease and Yersinia infections. Interestingly, these anti-citrullinated peptide antibodies were not found in malaria, syphilis, salmonella, chlamydia, legionella, streptococcus pyogenes, nor infectious endocarditis. It should be noted that these studies used different assays and so absolute comparisons are challenging. Nonetheless, not all infection appear to carry equal risk for ACPA positivity.

Some studies point to a relationship between ACPA-positive RA and periodontitis associated bacteria (27); other studies show associations specifically with *Porphyromonas gingivalis* (28–30). *P. gingivalis* is associated with periodontitis and described as a keystone pathogen capable of orchestrating microbial dysbiosis (31). *P. gingivalis* expresses a *P. gingivalis* Peptidyl Arginine Deiminase, (PPAD) that converts arginine residues to citrulline on both microbial and host proteins. Together with the combined expression of *P. gingivalis'* arginine gingipain, a protease capable of cleaving proteins at arginine peptide bonds, PPAD can generate non-endogenous C-terminal citrullinated peptides. The candidate autoantigens human fibrinogen and α-enolase were proteolytically cleaved and citrullinated following incubation with *P. gingivalis* (Wegner, 2010). There is a connection between *P. gingivalis*-mediated citrullination, inflammation in the gingivae and the subsequent generation of ACPA (28–30).

Whether anti-citrullinated autoimmunity can be triggered in the periodontal tissues and progress to joint inflammation consequent to failed mucosal compartmentalisation and evolution of the ACPA response remains to be determined.

## Antigen-processing and presentation

Data demonstrating that the periodontal tissues replicate the citrullinome of the arthritic joint (32), that oral bacteria are highly citrullinated (25), and the hypothesis that ACPA might evolve to bind citrullinated proteins at mucosal surfaces are intriguing (15), especially when considered in light of a recent study revealing that citrullination alters antigen-processing, leading to presentation of cryptic epitopes recognised by CD4+ T cells from patients with rheumatoid arthritis (33).

Previous experimental studies have revealed that T cells reactive with immunodominant self-epitopes are rendered tolerant, while T cells potentially reactive with immunorecessive or “cryptic” self-epitopes can escape tolerance (34, 35). During normal physiological self-antigen processing, cryptic epitopes are defined as those that are normally hidden from T cell immunosurveillance because they are not available to bind (or cannot bind) MHC complexes. However, under inflammatory conditions, modification of self-proteins, for example via post-translational modification, can change how self-proteins are processed by altering proteolytic cleavage, leading to the generation of new epitopes, or modified-epitopes with altered affinity for MHC complexes (36). Indeed, oxidative modifications introduced to the host cell proteome during metabolic stress are linked with changes in MHCII antigen-processing (2).

The study by Curran et al, reveal that citrullination of host proteins alters their antigenic processing giving rise to an alternative set of MHC epitopes compared with epitopes presented following processing of unmodified proteins. Since cryptic self-epitopes can have homology with microbial epitopes (37), and T cells reactive with cryptic self-epitopes can be engaged by immunisation with foreign antigen (38), it is conceivable that altered-antigen presentation following processing of modified proteins could underlie hypotheses relevant to bystander activation and molecular mimicry.

Notably, while some cryptic epitopes are implicated in the induction and propagation of pathogenic autoimmunity, others can help to down-regulate pathogenic autoimmune responses (36). In a model of adjuvant-induced arthritis (AA) in the Lewis rat, diversification of the T cell response to cryptic epitopes from mycobacterial 65 kDa heat-shock protein (Bhsp65), which are cross-reactive with a rat heat-shock protein, alleviate the course of AA (39). Furthermore, immunisation of Lewis rats with cryptic peptides derived from Bhsp65, protected mice from induction of AA following immunisation with *Mycobacterium tuberculosis* H37Ra (37). These data demonstrate that adaptive autoimmune responses can be engaged in both the propagation and regulation of autoimmune disease depending on the nature of the epitopes presented to autoreactive CD4+ T cells. Importantly, while in the Lewis rat induction of AA induces both arthritogenic and regulatory autoimmune responses, in a related strain of rat (Fischer 344) the course of AA can be modulated solely by exposure to environmental factors. Fischer rats raised in a barrier-facility are susceptible to AA, in contrast Fischer rats raised in a conventional facility had a reduced incidence of AA. Notably, naïve Fischer rats raised in the conventional facility demonstrated evidence of prior T cell activation towards regulatory cryptic epitopes derived from mycobacterial Bhsp65. This was attributed to spontaneous priming of T cells against regulatory epitopes of Bhsp65 from molecular homologues derived from microbes present in the conventional facility (40). These data demonstrate that environmental exposures can modulate the course and severity of autoimmune disease by determining whether the outcome of adaptive self-recognition is immunopathogenic or immunoregulatory.

## Functional consequences of antigen modification

Post-translational modification (PTM) poses a danger for the development of autoimmunity by changing how proteins are processed for antigen-presentation, potentially giving rise to cryptic (33) and/or modified-epitopes (2, 3) recognised by autoreactive T cells. Whereas cryptic epitopes are anticipated to be recognised as “non-self” and to recruit a different T cell population compared with T cells recruited by dominant self-epitopes, PTM-modified epitopes can be recognised as non-self (2, 3), or as we have shown, can change the interaction between antigen-presenting cells and antigen-specific CD4+ T cells, leading to altered functional responses (41).

CD4+ T cells from OTII mice express a transgenic T cell receptor specific for ovalbumin peptide^323–339^ (pOVA) and are useful for studying the interaction between antigen-presenting cells and functional outcomes in CD4+ T cells. Using this system, w modified the C-terminal arginine of pOVA to generate C-terminal citrullinated pOVA (pOVA-cit) to show that pOVA-cit changed the interaction between antigen-presenting cells and OTII T cells, leading to changes in T cell function in the context of immune-priming and immune-tolerance. Importantly, we demonstrated that OTII T cells responding to pOVA-cit were less dependent on co-stimulatory checkpoints for robust effector responses, compared with the OTII response to native pOVA. Further, using an oral tolerance model, we demonstrated that immunisation with pOVA-cit was sufficient to breach immune tolerance to native ovalbumin *in vivo*. This proof-of-concept study reveals that non-endogenous C-terminal citrullination can change the way CD4+ T cells “see” and respond to MHCII antigens.

The OTII TCR recognises a 9 amino acid core epitope (329–337) of pOVA (42). Therefore, the citrullinated residue (339) of pOVA: cit is predicted to be in the peptide-flanking region, and available to interact with the OTII TCR. Modification of flanking regions of MHCII peptides can determine CD4+ T cell functional outcomes, by selecting different populations of CD4+ T cells, or as predicted for pOVA: cit, by modifying the affinity of MHCII: peptide interactions with cognate TCRs (43, 44). Ultimately, whether endogenous or non-endogenous citrullination favours T cell autoimmunity will be dependent upon the T cell repertoire selected in the thymus, and at the site of inflammation.

## Discussion

The epidemiological relationship between periodontitis and RA well-established (45), and there is good evidence that periodontitis and infection with *P. gingivalis* are risk factors for ACPA positivity, at least in some patients. However, it remains to be determined whether ACPA positivity consequent to periodontitis, or *P. gingivalis* exposure favours transition to ACPA positive RA—and if so, whether this is based on genetic susceptibility, or other factors. Periodontitis is highly prevalent and a significant challenge for affected individuals, and its socioeconomic costs are substantial (46). Understanding the aberrant immune response in periodontitis has potential to improve treatment and prevention. Moreover, we propose that periodontitis could represent a platform to determine the causal pathways underlying the emergence and maturation of ACPA responses predisposing to RA. Broadly, there is an opportunity to understand fundamental and causal pathways of autoimmunity, autoimmune pre-disease, and perhaps the clinically manifest autoimmune disease. There is a significant clinical unmet need for this information. Patients with Rheumatoid arthritis (RA) have benefitted from transformative advances in therapies. RA disease remission (or relatively low disease activity) is achieved for more patients than ever. However, advanced therapies are expensive, can have significant side effects, and are not effective in every patient. Partial response and non-response still represent an unmet need, and therapy is usually lifelong therapy (47). The goal of drug-free remission remains elusive. RA still inflicts significant burdens on individuals and society. Annual direct costs (e.g., medications, medical and other care, adaptations) and indirect costs (e.g., lost productivity) are estimated at USD10,000–30,000 per patient, with some estimates up USD83,000 (48). Of patients treated with anti-TNF over 6 years who were consistently defined as “in remission” 57% of these patients report some compromised physical function (49). A study of 640 patients over 8 years found that 20% of RA patients who were defined as in remission reported Health Assessment Questionnaire (HAQ) scores > 1; indicating moderate to severe disability (50). The HAQ scores were significantly *higher* in patients with progressing RA who did not achieve remission. However, these and other studies (51) show that a sizable proportion of patients considered to achieve “good” outcomes still suffer. Understanding the biology in the mouth may offer transformative approaches to these significant challenges.
